# Pediatric neurosurgery training during residency in Switzerland and the need for dedicated subspecialization training

**DOI:** 10.1007/s00381-024-06343-6

**Published:** 2024-03-08

**Authors:** Ladina Greuter, Maria Licci, Raphael Guzman, Jehuda Soleman

**Affiliations:** 1 Department of Neurosurgery, University Hospital Basel, Spitalstrasse 21, 4031 Basel, Switzerland; 2 Division of Pediatric Neurosurgery, Children’s University Hospital of Basel, Basel, Switzerland; 3 Faculty of Medicine, University of Basel, Basel, Switzerland

**Keywords:** Pediatric neurosurgery, Neurosurgery residency, Subspeciality training, Pediatric neurosurgery training

## Abstract

**Introduction:**

Pediatric Neurosurgery as a subspeciality started to emerge during the late 1950s, with only a few dedicated pediatric neurosurgeons in the Western world. Over the last few decades, the awareness that children require subspecialized care by dedicated pediatric neurosurgeons and an interdisciplinary team has been growing worldwide, leading to an increase in pediatric neurosurgeons. Several studies have shown that subspecialized care for pediatric patients improves outcomes and is cost-effective. This survey aims to assess the current setting of pediatric neurosurgery and training of neurosurgical residents in pediatric neurosurgery in Switzerland.

**Methods:**

We conducted an online survey by sending e-mail invitations in 2021 to all neurosurgical residents in Switzerland. The survey included questions regarding the participants' demographics, current workplace structures, the care of specific pediatric neurosurgical pathologies, and participants’ opinions of the Swiss training program for pediatric neurosurgery and possible improvement. We defined at the beginning of the survey that a pediatric neurosurgeon is a board-certified neurosurgeon with at least one year of dedicated pediatric neurosurgical fellowship training abroad.

**Results:**

We received a total of 25 responses from residents, of which 20 (80%) were male. Twenty-two participants (88%) worked in one of seven major hospitals in Switzerland at the time of the survey, and four (16%) were interested in pursuing a fellowship in pediatric neurosurgery. Seven (35%) and five residents (25%) feel comfortable taking care on the ward of a craniosynostosis and hydrocephalus patient younger than 6 months, respectively. Twelve residents (60%) feel comfortable taking care of a pediatric brain tumor patient. The majority (n = 22, 88%) of all residents agree that a fellowship-trained pediatric neurosurgeon should treat children, while two (8%) residents state that any neurosurgeon with an interest in pediatric neurosurgery should be able to treat children. All residents (n = 25, 100%) agree that pediatric neurosurgery training and care in Switzerland needs to be improved.

**Conclusion:**

Pediatric neurosurgery training in Switzerland is rather heterogeneous and not very well structured, with varying frequencies of children-specific neurosurgical pathologies. Most residents agreed that a subspecialized pediatric neurosurgeon should oversee the care of children in neurosurgery, while all agree that pediatric neurosurgical training and care should be improved in Switzerland.

## Introduction

Pediatric neurosurgery as a subspeciality started to emerge during the late 1950s in the Western world, with only a few dedicated pediatric neurosurgeons caring for children [1]. Over the last few decades, the awareness that children require subspecialized care by dedicated pediatric neurosurgeons, and an interdisciplinary team has been growing worldwide, leading to an increase in pediatric neurosurgeons [2]. Several studies have shown that subspecialized care for pediatric patients is cost-effective and improves outcome [3–7]. The European Society for Pediatric Neurosurgery (ESPN) is the oldest society and was founded in 1967, followed by the International Society for Pediatric Neurosurgery (ISPN) in 1972 [1]. The latter currently has over 500 members all across the globe, and its mission is to improve the health and welfare of children requiring neurosurgical care throughout the world via scientific research and close international cooperation [8]. In 1991 the American Board of Pediatric Neurological Surgery was founded to provide subspecialty accreditation, including accreditation of fellowship programs and board examinations [9]. Similarly, in the UK, a dedicated pediatric neurosurgical fellowship is mandatory to provide neurosurgical care to children. However, in other parts of the world, including many European, Latin American, and Asian countries, structured fellowship programs as well as dedicated sub-specialization regulations are scarce or do not exist [2, 10, 11]. In Switzerland, historically, pediatric neurosurgical diseases such as hydrocephalus, craniosynostosis, and even dysraphism were, and in some institutions still are, conducted by general pediatric surgeons, while pediatric brain tumors are operated by “adult” neurosurgeons. The Children’s University Hospital of Basel was the first Children’s Hospital in Switzerland which defined that all neurosurgical diseases in children are to be managed and operated under the care of a fellowship-trained pediatric neurosurgeon. Simultaneously, an educational and training program for residents and fellows dedicated in pediatric neurosurgery was established and is presently run by four fellowship-trained pediatric neurosurgeons.

The aim of this survey is to assess the current setting of pediatric neurosurgery and the training of neurosurgical residents in pediatric neurosurgery in Switzerland.

## Methods

We conducted an online survey by sending e-mail invitations to all neurosurgical residents in Switzerland. To ensure a thorough distribution, we also distributed the survey via the Swiss Young Neurosurgeons (SYNS) association. The SYNS has approximately 80 members which are residents in Switzerland. The online survey consisted of a total of 44 questions and was prepared and launched via Google Forms (Google LLC, Mountain View, CA, USA), which is a web-based survey platform. The questionnaire contained four questions about the participants’ demographics and four questions about the structure of their current workplace. Further, we asked questions regarding the care of four specific pediatric neurosurgical pathologies: craniosynostosis, hydrocephalus, tethered cord, and brain tumors. We dichotomized tethered cord into simple tethered cord (e.g., in case of a filar lipoma) and lipomyelomeningocele, since these surgeries differ quite significantly from one another. The last six questions were about the participants’ opinion regarding the Swiss pediatric training program and possible improvement. We defined at the beginning of the survey that a pediatric neurosurgeon is a board-certified neurosurgeon with at least 1 year of dedicated pediatric neurosurgical fellowship training abroad. The e-mails were first sent out in July 2021 and a reminder e-mail was sent out 6 months later (Table 1). The participation rate is estimated at 35% given the number of neurosurgical residents in the country.

**Table 1:** Questions of the survey with possible answers

**Survey pediatric neurosurgery**	
What is your gender?	Female, Male, prefer not to say
What is your age in years?	< 20, 20–25, 26–30, 31–35, 36–40, > 40
Which year of residency are you in? (Minimal training requirement is 6 years)	1–8
Which type of teaching hospital are you working in?	University hospital Regional hospital Local hospital
How many pediatric cases are treated at your department in a year?	0, < 20, 20–40, 40–60, 60–80, 80–100, 100–120, 120–140. 140–160, > 160
Is there an outpatient clinic for pediatric neurosurgery in your clinic?	Yes, no, maybe
Are there interdisciplinary boards/clinics (tumor, spasticity, dysraphism, phacomatosis, etc.) discussing pediatric neurosurgery cases in your clinic?	Yes, no, maybe
Is there a fellowship-trained pediatric neurosurgeon working in your department?	Yes, no, maybe
How many craniosynostosis do you treat at your current department per year?	0, < 5, 5–10, 10–15, 15–20, 25–30, 30–40, 40–50, > 50
Have you ever seen/treated a craniosynostosis in the ward or outpatient clinic at your current department?	Yes, no
Have you ever seen/treated a craniosynostosis in the operating room at your current department?	Yes, no
Who mainly performs craniosynostosis surgery at your current department?	pNS, NS, PS, pNS and NS, pNS and PS,NS and PS, I do not know
Would you feel comfortable and capable taking care of a craniosynostosis patient?	Yes, no
How many pediatric hydrocephalus patients do you treat at your current department per year?	0, < 5, 5–10, 10–15, 15–20, 25–30, 30–40, 40–50, > 50
Have you ever seen/treated a pediatric hydrocephalus patient in the ward or outpatient clinic at your current department?	Yes, no
Have you ever seen/performed a neuroendoscopy for pediatric hydrocephalus in the operating room at your current department?	Yes, no
Have you ever seen/performed a shunt for pediatric hydrocephalus in the operating room at your current department?	Yes, no
Have you ever seen/performed a pediatric hydrocephalus procedure in a baby under the age of 6 months at your current department?	Yes, no
Who performs pediatric shunt surgeries at your current department?	pNS, NS, PS, pNS and NS, pNS and PS, NS and PS, pNS cranial-PS abdominal, NS cranial-PS abdominal I do not know
Who mainly performs pediatric neuroendoscopic surgeries at your current department?	pNS, NS, PS, pNS and NS, pNS and PS, NS and PS, I do not know
Would you feel comfortable and capable taking care of a pediatric hydrocephalus patient?	Yes, no
Would you feel comfortable and capable taking care of a pediatric hydrocephalus patient under the age of 6 months?	Yes, no
How many tethered cord patients (incl. filar lipoma) do you treat at your current department per year?	0, < 5, 5–10, 10–15, 15–20, > 20
How many patients with a lipomyelomeningocele do you treat at your current department per year?	0, < 5, 5–10, 10–15, 15–20, > 20
Have you ever seen/treated a tethered cord (incl. filar lipoma) patient on the ward or outpatient clinic at your current department?	Yes, no
Have you ever seen/treated a patient with a lipomyelomeningocele on the ward or outpatient clinic at your current department?	Yes, no
Have you ever seen/performed an untethering surgery in the operating room at your current department?	Yes, no
Have you ever seen/performed an untethering surgery for lipomyelomeningocele in the operating room at your current department?	Yes, no
Who mainly performs tethered cord surgeries (incl. filar lipoma) at your current department?	pNS, NS, PS, pNS and NS, pNS and PS, NS and PS, I do not know
Who mainly performs untethering surgeries for lipomyelomeningocele at your current department?	pNS, NS, PS, pNS and NS, pNS and PS, NS and PS, I do not know
Would you feel comfortable and capable taking care of a tethered cord patient?	Yes, no
Would you feel comfortable and capable taking care of a patient with lipomyelomeningocele?	Yes, no
How many patients with a pediatric brain tumor do you treat at your current department per year?	0, < 5, 5–10, 10–15, 15–20, 25–30, 30–35, 35–40, 40–45, 45–50, > 50
Have you ever seen/treated a patient with a pediatric brain tumor on the ward or outpatient clinic at your current department?	Yes, no
Have you ever seen/performed a pediatric brain tumor resection in the operating room at your current department?	Yes, no
Who mainly performs pediatric brain tumor surgeries at your current department?	pNS, NS, PS, pNS and NS, pNS and PS, NS and PS, I do not know
Would you feel comfortable and capable taking care of a patient with a pediatric brain tumor?	Yes, no
Based on your overall experience in neurosurgery have you had the chance to gain experience in pediatric neurosurgery outside of your center?	Yes another Swiss hospital, yes abroad, no
If you said “yes, abroad” in the question above, please state in which country you gained your experience?	Free text
Based on your personal opinion does education in pediatric neurosurgery need to be improved in Switzerland?	Yes, no
Based on your personal opinion who do you think should treat children with neurosurgical diseases?	Fellowship-trained pNS, any NS with interest in pediatric neurosurgery, any PS with interest in neurosurgery, depends on pathology, I do not care, I do not know
Are you personally interested in pediatric neurosurgery?	Yes, no, maybe
Are you planning on pursuing a career specializing in pediatric neurosurgery?	Yes, no, maybe
Comments	Free text

*pNS* pediatric neurosurgeon, *NS* general neurosurgeon, *PS* pediatric surgeon

All data was collected automatically in Google Forms and exported to the statistical software R (Version 1.3.1093, R Foundation for Statistical Computing, Vienna, Austria). The data was anonymous, but the participants could submit their e-mail addresses. Descriptive statistics were conducted for all responses.

## Results

### Baseline demographics and workplace characteristics

We included a total of 25 responses, of which 20 (80%) were from male residents. Over half of the residents (*n* = 13, 52.0%) were 26–30 years of age, followed by ten residents (40%) between 31 and 35 years, and only two participants (8%) were over 35 years of age. Most residents (*n* = 15, 72%) were in the second half of their residency training (years 4–6). Twenty-two participants (88%) were working in an academic institution at the time of the survey. Of the participating residents, 60% (*n* = 15) have a personal interest in pediatric neurosurgery, with four (16%) planning to pursue a career in this field and 32% (*n* = 8) who were undecided if they want to subspecialize in pediatric neurosurgery.

**Fig. 1:**
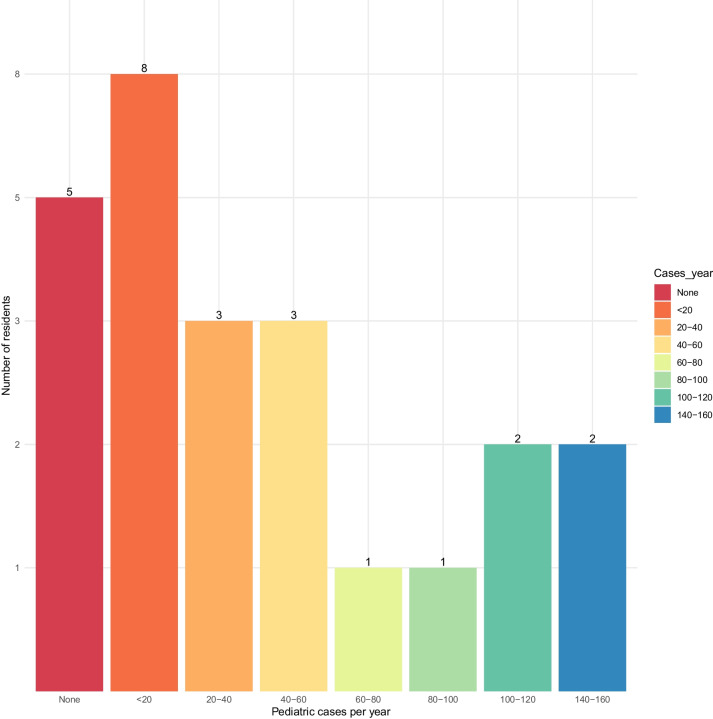
Number of all pediatric neurosurgery cases operated on per annum in the residents’ current workplace

Five residents (20%) reported working at an institution where no pediatric neurosurgical cases are performed, and they subsequently did not answer the questions regarding specific pathologies. The distribution of overall pediatric neurosurgical cases per year at the residents’ institutions was heterogeneous (Fig. 1). Fifteen (60%) residents reported that their workplace does not have a dedicated pediatric neurosurgical outpatient clinic. However, 22 (52%) of the participating residents stated that there are pediatric multidisciplinary team (MDT) meetings at their hospital. Only seven (28%) of all residents worked in a hospital with a fellowship-trained pediatric neurosurgeon. Centers with a fellowship-trained pediatric neurosurgeon operated on significantly more cases per year compared to centers without it (*p* = 0.003). Further, centers with a pediatric neurosurgeon had a higher rate of a dedicated outpatient clinic compared to the others (*p* = 0.001).

For several pathologies, approximately a third (21–33%) of all participants did not know if a dedicated pediatric neurosurgeon was responsible for the care of the children or if it was either an “adult” neurosurgeon or a pediatric surgeon. Only 36.8% (*n* = 7) of the residents reported consistently that a pediatric neurosurgeon would be responsible.

### The treatment of specific pediatric neurosurgical pathologies

#### Craniosynostosis

**Fig. 2:**
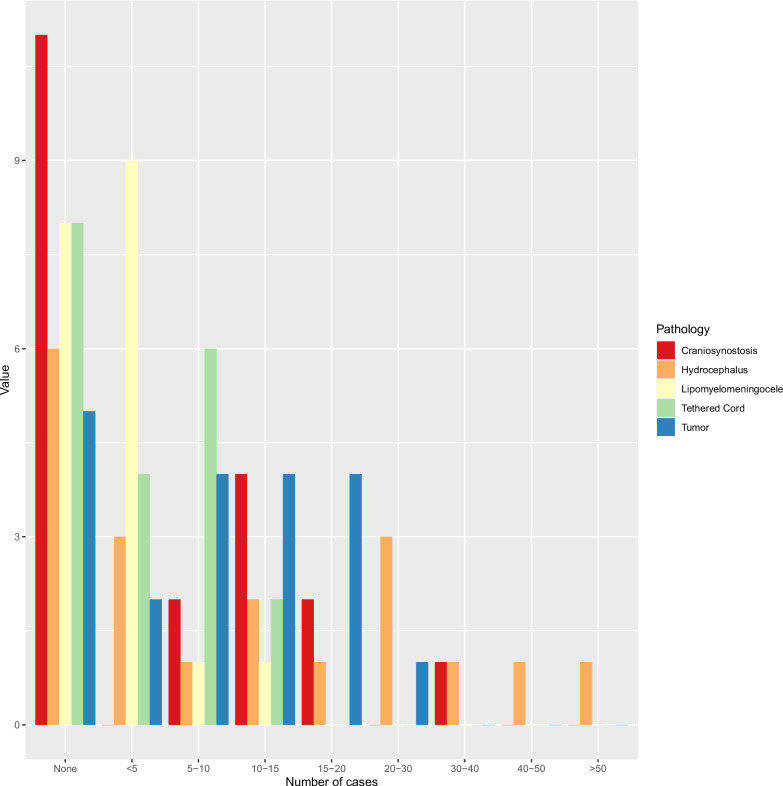
Number of the different pathologies treated per annum in the residents’ current workplace

**Fig. 3:**
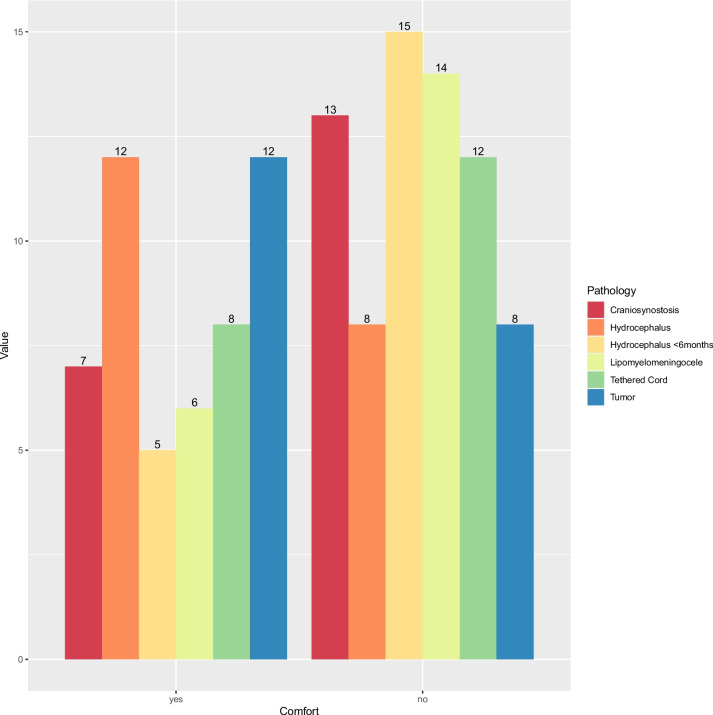
The number of residents feeling comfortable treating pediatric neurosurgical pathologies

Over half of the participating residents (*n* = 11, 55%) reported that craniosynostosis in general was not treated at their institution (Fig. 2). Eight (40%) residents have been treating patients with craniosynostosis on the ward or in an outpatient clinic. Six (30%) have been involved in the operative treatment of craniosynostosis. Six (30%) residents were not sure who was mainly treating these patients in their institution, while seven (35%) residents reported that pediatric neurosurgeons were treating craniosynostosis. Two residents (10%) stated that general pediatric surgeons were treating craniosynostosis at their hospital. Thirteen residents (65%) do not feel comfortable taking care of craniosynostosis patients (Fig. 3). Residents who trained at centers with fellowship-trained neurosurgeons felt significantly more comfortable treating children with craniosynostosis (*p* = 0.04).

#### Hydrocephalus

Pediatric hydrocephalus was not treated in a third (*n* = 6, 30%) of the participants’ hospitals, while half of the residents (*n* = 10, 50%) reported that less than 30 cases were treated at their hospital per annum (Fig. 2). Nine residents (45%) have seen/performed a neuroendoscopy for pediatric hydrocephalus, while 11 residents (55%) have been involved in a ventriculoperitoneal shunt (VPS) surgery for children. Eight residents (40%) have been part of a pediatric hydrocephalus surgery in a patient < 6 months of age. In 45% (*n* = 9) of all responses, pediatric neurosurgeons are mainly performing VPS or neuroendoscopic surgeries in their department, while two residents (10%) reported that during VPS surgery, the cranial part was performed by pediatric neurosurgeons while the abdominal part was done laparoscopically by general pediatric surgeons. One resident (5%) reported that general pediatric surgeons performed the whole procedure (VPS and neuroendoscopy) without any neurosurgical input. Sixty percent (*n* = 12) of all respondents would feel comfortable taking care of a pediatric hydrocephalus patient, while only 25% (*n* = 5) feel this way in a patient < 6 months of age (Fig. 3). No significant difference regarding the comfort levels of treating children with hydrocephalus was observed between the residents training at a center with a fellowship-trained pediatric neurosurgeon compared to the rest. However, significantly more residents have taken care of such patients in the ward/clinic or operating room in centers with a fellowship-trained pediatric neurosurgeon (*p* = 0.01).

#### Tethered cord/lipomyelomeningocele

Tethered cord surgeries were reported to be the least performed per year in any hospital, according to the residents (Fig. 2). Eleven (55%) and 13 (65%) residents have never treated a patient with all types of tethered cord in the ward and outpatient clinic, respectively. Similarly, only seven (35%) and five (25%) residents have been involved in the operating room in a tethered cord and lipomyelomeningocele surgery, respectively. In 45% (*n* = 9) of all responses, residents indicated that mainly pediatric neurosurgeons treat tethered cord and lipomyelomeningoceles, while one resident each (5%) stated that either adult and pediatric neurosurgeons, or adult neurosurgeons alone, and pediatric surgeons alone treat these patients. Sixty percent (*n* = 12) and 70% (*n* = 14) of the participating residents do not feel comfortable taking care of tethered cord and lipomyelomeningoceles patients, respectively (Fig. 3). Significantly more residents have treated patients with tethered cord or lipomyelomeningoceles in centers with a fellowship-trained pediatric neurosurgeon compared to others (*p* = 0.04).

#### Brain tumors

Brain tumor surgeries were reported to be some of the most common pediatric neurosurgeries, with four residents (20%) each reporting that their current workplace treats either 5–10, 10–15, or 15–20 patients per year (Fig. 2). One resident (5%) reported that 25–30 brain tumors were treated at his/her current workplace. Seventy percent (*n* = 14) of all residents have either been involved in the outpatient or ward care of pediatric brain tumor patients, as well as in the operating room. Half of all the participating residents reported that mainly pediatric neurosurgeons are treating these patients, while two residents (10%) reported that either adult neurosurgeons alone or adult and pediatric neurosurgeons would operate on children with brain tumors. Sixty percent (*n* = 12) of all residents feel comfortable taking care of these children (Fig. 3). No significant difference was observed between centers with a fellowship-trained pediatric neurosurgeon compared to other centers regarding the comfort level of residents.

### Pediatric neurosurgical training

**Fig. 4:**
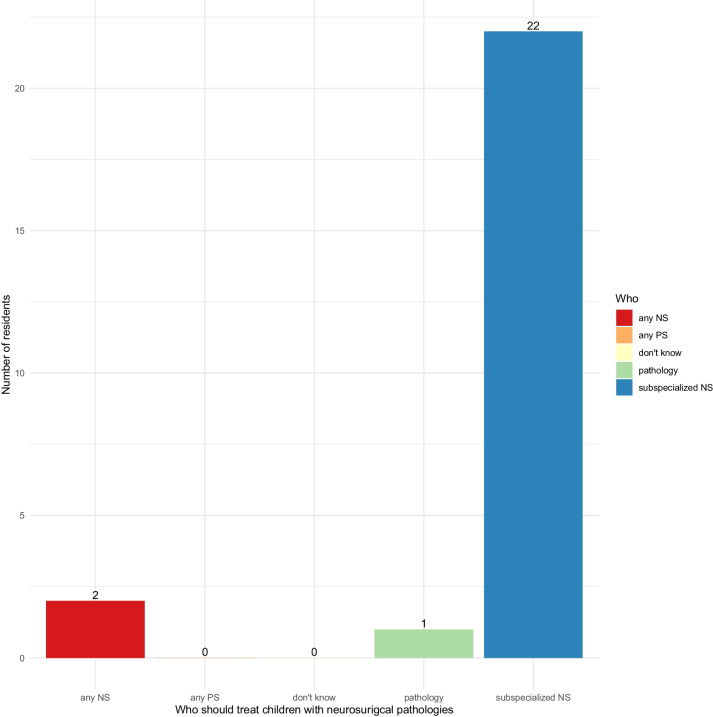
Who should treat children with neurosurgical pathologies according to the participating residents (NS, any neurosurgeon with an interest in pediatric neurosurgery; PS, any pediatric surgeon with an interest in neurosurgery; pathology, depending on specific pathology)

Twelve residents (48%) were not able to gain more training in pediatric neurosurgery outside their current workplace, while nine (36%) had exposure to pediatric neurosurgery at another Swiss institution, and four (16%) residents reported that they gained experience in pediatric neurosurgery abroad. The residents who gained experience abroad were one (4%) in Canada, one (4%) in France, and two (8%) in Italy. The majority (*n* = 22, 88%) of all residents agree, that a fellowship-trained pediatric neurosurgeon should treat children. Two (8%) residents state that any neurosurgeon with an interest in pediatric neurosurgery should be able to treat children, and one resident (4%) thinks that the treatment should be dependent on the pathology and indicates that brain tumors should be treated by neurosurgeons while the remaining pathologies should be treated by general pediatric surgeons (Fig. 4). All residents (*n* = 25, 100%) agree that pediatric neurosurgery training in Switzerland needs to be improved.

## Discussion

Based on the present survey conducted amongst approximately a third of the residents in Switzerland, 72% reported that no fellowship-trained pediatric neurosurgeon is working at their institution, even though 88% of the responders are working in one of the main academic hospitals in Switzerland. Further, pediatric neurosurgical diseases are often treated by adult neurosurgeons or even general pediatric surgeons, probably explaining the limited exposure and case load of common pediatric neurosurgical diseases in most institutes. The exposure of residents to the treatment of pediatric hydrocephalus (70%) and pediatric brain tumors (75%) was higher than for craniosynostosis (45%) and dysraphism (40%), which was often limited to centers with fellowship-trained pediatric neurosurgeons. To note, that 16% of the residents gained experience in pediatric neurosurgery outside of Switzerland. Overall, the exposure to pediatric neurosurgery during residency in Switzerland seems to be rather minimal, and all residents agreed that the pediatric neurosurgery training should be improved.

### The subspeciality of pediatric neurosurgery: Historical aspects

Historically, children with neurosurgical diseases were mainly treated by pediatric surgeons or in some exceptions (e.g., pediatric brain tumors) by adult neurosurgeons. In the 1950s the neurosurgical community felt a need for subspecialized pediatric neurosurgeons [1]. At that time, a few institutions amongst others London, Boston, Toronto, Paris, and Buenos Aires began to introduce dedicated pediatric neurosurgical care by subspecialized neurosurgeons [1]. However, the foundation of a pediatric subspeciality was not always welcomed by all members of the neurosurgical society.

The first dedicated society for pediatric neurosurgery was the European Society for Pediatric Neurosurgery which was founded in 1967. Subsequently, the International Society of Pediatric Neurosurgery (ISPN) was founded in 1972 with founding members from Argentina, Canada, the USA, the UK, Norway, France, Austria, and Japan [12]. This was followed shortly after in 1978 with the foundation of the American Society of Pediatric Neurosurgery. The American Board of Pediatric Neurological Surgery was the first to introduce subspecialty accreditation, including an accredited fellowship program and board exam in 1991 [9]. So far this remains the only national pediatric neurosurgical board exam. Other countries, such as the UK pre-requisites fellowship training to practice as a pediatric neurosurgeon. Despite this early advent of specialized pediatric care in Europe and both North and South America, dedicated pediatric neurosurgical care only emerged a few decades later in Asia [2]. One of the largest centers in Asia is the Tiantan Hospital Neurosurgery Center in Bejing, where a dedicated pediatric neurosurgical unit was founded in the 1980s [13]. Japan founded its own Japanese Pediatric Neurosurgical Society in the 1970s and the pediatric sub-specialization had its advent after the ISPN meeting, which took place in Tokyo in 1973 [14].

### Pediatric neurosurgical patients are not small adult neurosurgical patients

There used to be amongst neurosurgeons a certain belief that pediatric neurosurgery is general neurosurgery on small adults [15]. Our group, along with many other groups, strongly believes that children should be cared for by fellowship-trained pediatric neurosurgeon and not by an adult neurosurgeon or a pediatric surgeon. The differences in physiology such as body weight, blood volume, and metabolism require specific attention and a trained interaction between the surgeon and the pediatric anesthesiologist. The same and sometimes seemingly simple pathologies in children and adults, such as hydrocephalus or brain tumors, often have significantly different etiologies, pathophysiology, and treatment philosophies in the two age groups [16–21]. There is no doubt that a skilled adult neurosurgeon can technically perform successful surgery on children; however, a pediatric neurosurgeon should be defined as more than just a skilled mechanic operating on children, but additionally incorporate a different treatment philosophy often required in children. Further, there are several pediatric neurosurgical pathologies, such as craniosynostosis or spinal dysraphism, a variety of genetic syndromes, and surgical skills that have no counterpart in general or adult neurosurgery, as well as in general pediatric surgery. Lastly, children, as opposed to adults, frequently require a multidisciplinary care setting that requires subspecialized knowledge and often multidisciplinary-led clinics such as a spina bifida, spasticity, or craniofacial clinic, involving many different subspecialties within a children’s hospital.

### Pediatric neurosurgery in Switzerland and beyond: Availability, training, and future directions

To date, the treatment of pediatric patients with neurosurgical diseases in Switzerland is very heterogenous and not in the hands of subspecialized pediatric neurosurgeons in the majority of the centers. The present survey underlines this fact and suggests an overall lack of training and exposure of residents to pediatric neurosurgery in Switzerland. Many pediatric patients with neurosurgical diseases are either not cared for in their institutions or are cared for by pediatric surgeons or adult neurosurgeons. This probably leads to a large difference and variety in the treatment of pediatric neurosurgical diseases amongst centers within Switzerland, ranging from no pediatric cases up to around 200 surgeries per year. Around 70% of all participating residents reported that there is no fellowship-trained pediatric neurosurgeon in their current workplace, but 88% felt that a fellowship-trained pediatric neurosurgeon should oversee the care of children in neurosurgery. All participating residents agreed that pediatric neurosurgical training should be improved in Switzerland. Currently, there is no dedicated pediatric neurosurgical training in Switzerland. There is no mandatory census of pediatric neurosurgical cases in the logbook for board certification. Further, to date, there are no clear guidelines or laws that children should be treated by a subspecialized pediatric neurosurgeon. Given these circumstances, there is a concern that specific aspects of pediatric neurosurgery are not adequately covered during residency in Switzerland, while some aspects of pediatric neurosurgery might even be under-treated in some regions within Switzerland. Extrapolating from the countries, where pediatric neurosurgery programs are established, some measures could be defined to improve the lack of education in pediatric neurosurgery amongst Swiss residents and generally improve the care of pediatric neurosurgical patients. First, a subspecialized board certification for pediatric neurosurgery could be established, defining the qualifications one would need to become a pediatric neurosurgeon. This would include a specific training time within a pediatric neurosurgical center (in Switzerland and/or abroad), education in pediatric neurosurgery (e.g., courses and conferences), as well as completing a surgical logbook. Second, the healthcare legislation should be changed so that pediatric neurosurgical diseases can only be cared for by specialized pediatric neurosurgeons and not by general pediatric surgeons or adult neurosurgeons. Centralization of pediatric neurosurgical care in larger hospitals, where a specialized pediatric neurosurgical team is an integral part of the Children’s hospital, is required to obtain a certain caseload of these rare pathologies, to offer thoroughly specialized care and adequate training. Finally, educating the local communities and general pediatricians on the existence, importance and need to refer these patients to specialized pediatric neurosurgeons offer streamlined referral pathways. Similar concerns were and still are present in other parts of the world. This led to the foundation of national pediatric neurosurgical training and fellowship programs in some countries [10, 22]. The main goal of any pediatric fellowship is to improve the care of children with neurosurgical diseases. This implies that a fellowship should expose the trainees to a large number of different pediatric neurosurgical pathologies and up-to-date care [22]. Our group strongly agrees that sub-specialization is paramount and that pediatric neurosurgery requires a different subset of knowledge, skills, and philosophy than general neurosurgery, which can only be accomplished by completing a defined program including a fellowship in pediatric neurosurgery.

In our survey, only 16% of all participating residents planned to pursue a career in pediatric neurosurgery, while 32% were undecided. The heterogenous exposure to pediatric neurosurgery during training could explain this rather large percentage of undecided residents and makes it difficult to determine the factors why residents choose to subspecialize in pediatric neurosurgery. A study that evaluated the American fellowship program after 15 years of its inception found no correlation between the different residency programs and the decision to seek pediatric neurosurgical sub-specialization [9]. The same paper by Durham et al. assumed that 8% of all neurosurgical residents subspecialize in pediatric neurosurgery, which would lead to a shortage of qualified neurosurgeons in pediatric neurosurgery, while other subspecialties, such as spine surgery, show a large increase in their training numbers [9, 23]. Globally, a recent survey showed that in the USA 5–6 pediatric neurosurgeons per 1,000,000 children were available, which is higher than in most parts of the world. The same survey showed that Switzerland’s density of pediatric neurosurgeons was 2–3 per 1,000,000, which is similar to Germany, France, Australia, Brazil, and Mexico. The highest density of pediatric neurosurgeons was found in Spain, Scandinavia, Russia, Chile, and Argentina [11]. However, given that these results are based on a survey distributed within pediatric neurosurgical societies, not clearly defining the qualifications of a pediatric neurosurgeon, a certain bias of the results cannot be excluded.

Several studies showed that subspecialized care was overall more cost-efficient and had a lower complication rate [3, 5, 24, 25]. Specifically for pediatric neurosurgery, a large study with 732 children analyzing the extent of resection in malignant pediatric brain tumors between adult and pediatric neurosurgeons showed a significantly higher extent of resection, which is known to influence outcome and survival, for fellowship-trained pediatric neurosurgeons [26, 27]. Another North American study showed that the mortality rates for pediatric brain tumor surgery were lower in larger centers, where the surgeons had a higher caseload [7]. Both these studies advocate the need for sub-specialization and centralization of pediatric brain tumor surgeries given the low overall incidence of these pathologies [7, 26]. We strongly agree that not onluy pediatric brain tumors but also other complex pathologies such as pediatric hydrocephalus, craniosynostosis, or tethered cord, which are rarely seen in the adult population, should be treated by pediatric neurosurgeons, which are part of interdisciplinary teams within a children’s hospital [16–21].

### Limitations

This study is a survey and is inherent to all limitations of such a study format. First, the presumed low participation rate (approximately 30% of all residents) and the self-reported data represent an unavoidable limitation. A low response rate raises the question of whether the survey is fully representative of the neurosurgical resident community. Given the low number of participating residents, no comparative statistics were conducted. To keep the survey as anonymous as possible, we did not assess the exact centers the respondents were working in; therefore, we cannot guarantee an equal distribution of responders between the centers. Also, it is required to rotate between different hospitals throughout the residency in Switzerland, and despite the survey being specifically focused on the residents’ current workplace, some of their experience and answers could be influenced by a previous rotation in a different hospital, even outside of Switzerland. Further, Switzerland has a population of approximately 8 million people, which leads to a limited number of children and hence to a low number of overall pediatric neurosurgical cases per year.

## Conclusion

Pediatric neurosurgery training in Switzerland is very heterogeneous, with varying frequencies of children-specific neurosurgical pathologies. Only very few hospitals have one or more fellowship-trained neurosurgeons with a dedicated pediatric neurosurgical training program. Most residents agreed that a subspecialized pediatric neurosurgeon should oversee the care of children in neurosurgery, while all agreed that pediatric neurosurgical training should be improved in Switzerland.

## Data Availability

Data can be requested directly from the corresponding author
